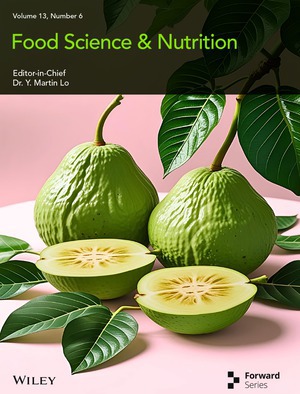# Cover Image

**DOI:** 10.1002/fsn3.70619

**Published:** 2025-08-28

**Authors:** Litun Ahmed Labib, Swagata Dey, Md. Fakhrul Hasan

## Abstract

The cover image is based on the article *Improving Guava Shelf Life and Preserving Postharvest Quality With Edible Coatings* by Litun Ahmed Labib et al., https://doi.org/10.1002/fsn3.70491.